# Natural Functional Beverages as an Approach to Manage Diabetes

**DOI:** 10.3390/ijms242316977

**Published:** 2023-11-30

**Authors:** Filomena Carvalho, Radhia Aitfella Lahlou, Paula Pires, Manuel Salgado, Luís R. Silva

**Affiliations:** 1 CPIRN-UDI/IPG—Centro de Potencial e Inovação em Recursos Naturais, Unidade de Investigação para o Desenvolvimento do Interior do Instituto Politécnico da Guarda, 6300-559 Guarda, Portugal; filomenacarvalho@ipg.pt (F.C.); radhialahlou@ipg.pt (R.A.L.); paulapires@ipg.pt (P.P.); vp_manuelsalgado@ipg.pt (M.S.); 2 CICS-UBI—Health Sciences Research Center, University of Beira Interior, 6201-506 Covilhã, Portugal; 3 CIEPQPF—Chemical Process Engineering and Forest Products Research Centre, Department of Chemical Engineering, University of Coimbra, Rua Sílvio Lima, Pólo II—Pinhal de Marrocos, 3030-790 Coimbra, Portugal

**Keywords:** diabetes mellitus, functional beverages, fruits, vegetables, phenolic compounds, anti-diabetic activity

## Abstract

Diabetes mellitus is a chronic disease, commonly associated with unhealthy habits and obesity, and it is becoming a serious health issue worldwide. As a result, new approaches to treat diabetes are required, and a movement towards more natural approaches is emerging. Consuming fruit and vegetables is advised to prevent diabetes since they contain several bioactive compounds. A simple and effective strategy to include them in the diets of diabetic and obese people is through beverages. This review aims to report the anti-diabetic potentials of different vegetable and fruit beverages. These functional beverages demonstrated in vitro potential to inhibit *α*-glucosidase and *α*-amylase enzymes and to improve glucose uptake. In vivo, beverage consumption showed a reduction of blood glucose, increase of insulin tolerance, improvement of lipid profile, control of obesity, and reduction of oxidative stress. This suggests the potential of vegetable- and fruit-based functional beverages to be used as a natural innovative therapy for the management of diabetes.

## 1. Introduction

Over the last few years, the market for functional foods has been growing and developing very quickly due to the increased prevalence of lifestyle diseases and awareness of the importance of a healthy lifestyle by the general public [1].

Chronic diseases are long-lasting illnesses that can be caused by a combination of genetic, physiological, environmental, and behavioural factors. They include cardiovascular diseases, cancers, chronic respiratory diseases, and diabetes [2]. Chronic diseases can be prevented and controlled but cannot be cured [3]. According to the World Health Organization (WHO), maintaining a healthy diet throughout one’s life helps prevent these non-communicable diseases, which are the leading causes of death worldwide [4].

The prevalence of diabetes has globally increased, rising from 4.7 in 1980 to 8.5% in 2014, with approximately 422 million people diagnosed with the disease in that year [5]. As of 2021, over half a billion people were living with diabetes [6]. The association between diet and chronic disease is well known [7,8,9]. In particular, diets low in the glycaemic index and carbohydrates and high in vegetables are associated with a more effective control of type 2 diabetes (T2D) [10].

The consumption of functional foods can be a good approach to promote healthy habits due to their ready availability and disease-preventing characteristics [3]. There are many different types of functional foods on the market right now including dairy foods, baked goods and cereals, baby foods, confectionary and meat products, snacks and readymade meals, spreads, and beverages. The benefits of functional beverages include the ability to add desired bioactive components and reduce production costs as well as the simplicity in the handling of the cold chain during storage and greatest acceptance by consumers [11].

Fruits and vegetables, which can be part of those functional beverages, contain many bioactive compounds including polyphenols, vitamins, minerals, and pectins, which stimulate insulin secretion, successfully reduce the glucose level of blood, and are also able to inhibit carbohydrate absorption in the small intestine [12,13,14,15]. The United States Department of Agriculture (USDA) recommends five to nine servings per day, while the WHO recommends a minimum of 400 g of fruits and vegetables every day [16]. Functional beverages developed from different fruit and vegetable blends align well with consumer expectations for unique yet nutritious products [17]. Hence, this current work reviews the scientific advancements made over the past 13 years, concerning the uses of functional beverages made from fruits and vegetables in addressing the chronic condition of diabetes.

## 2. Diabetes

Diabetes is a serious public health problem with a negative impact on socioeconomic development, mortality, and disability on a global scale [18]. In line with the 2011 Political Declaration on the Prevention and Control of NCDs, diabetes is one of the top five noncommunicable illnesses (NCDs) that needs to be controlled [19]. Diabetes affected 537 million people worldwide in 2021, and 700 million people are predicted to have the disease by 2045 [20]. It is a chronic condition characterized by elevated levels of blood glucose (hyperglycaemia), which gradually leads to severe damage to the heart, veins, eyes, kidneys, and nerves [5]. Insulin is responsible for the removal of glucose from the blood by liver and muscles cells, which is followed by glucogenesis [21]. Thus, this dangerous, chronic condition develops either when the pancreas fail to produce enough insulin or when the body is incapable of using the produced insulin effectively [22].

### 2.1. Type 1 Diabetes

Type 1 diabetes (T1D) is characterized by deficient insulin production in the body. To control the level of glucose in their blood, people with T1D need to take insulin daily; they cannot live without it [5]. The destruction of pancreatic *β*-cells is what leads to this deficit in insulin production, which may be acquired or epigenetic (autoimmunity/mutation) [23]. Aside from frequent urination and thirst, other symptoms include excessive hunger, weight loss, vision problems, and exhaustion [5]. The risk factors for this type of diabetes include autoimmune, genetic, and environmental factors, and as of now, there is no known method of prevention [24]. Children and teenagers are the most affected by T1D [5]. The European Region has the largest number of children and teenagers with T1D, totalling over 296,500 [25,26].

### 2.2. Type 2 Diabetes

T2D is perceived as a lifestyle-related disease since it often results from obesity, poor eating habits, and stress and is accountable for more than 90% of cases of diabetes [27]. T2D is additionally associated with a prolonged increase in blood glucose levels, which may have two causes: deficiencies in insulin secretion due to impairment of *β*-cell function or that of its receptor (insulin resistance) [28]. T2D patients may require exogenous insulin, even if they do not need it frequently, when diet and oral hypoglycaemic medications prove to be insufficient in controlling blood glucose levels. The symptoms of T2D are similar to those of type 1, however they are frequently absent or less pronounced, leading to more challenges [5]. According to WHO, the greater risk factor for developing T2D is being overweight or obese. A poor diet, inactivity, high blood pressure, and a family history of diabetes are also typical determinants of the disease [5]. Patients with T2D are prone to various immediate and long-term complications including malignancies, microvascular disorders (retinopathy, nephropathy, and neuropathy), and macrovascular diseases (hypertension, hyperlipidaemia, heart attacks, coronary artery disease, strokes, and peripheral vascular disease) [29].

### 2.3. Prevalence

Over the past few decades, there has been an increase in the prevalence of T2D worldwide. This is primarily as a result of sedentarism and a decrease in the nutritional values of diets, which has led to an increase in overweightness and obesity [30]. Over 1 million deaths were brought on by this illness in 2017.

In the European Region, approximately 60 million people have diabetes, with around 10.3% of men and 9.6% of women aged 25 years and over being affected [31]. In Portugal, there were more than 1 million diabetic people between the ages of 20 and 79 in 2018, indicating a prevalence of 13.6%.

### 2.4. Diagnosis and Treatment

Early diagnosis (thorough the measurement of HbA1C values, fasting blood sugar, or random blood sugar tests, according to the Centres for Disease Control and Prevention (CDC) guidelines [32]) and control of blood sugar, blood pressure, and cholesterol management can help prevent or delay the associated complications of diabetes [24].

Patients with T2D must follow a low-calorie diet and engage in regular physical activity, otherwise pharmaceutical therapy might be necessary [33]. In the current market, few therapeutic drugs have been introduced to regulate blood glucose metabolism to a normal state. The cost of treating diabetes and its complications is unpredictably high [34]. Particularly, the treatment plans used to try to manage diabetes are the use of hypoglycaemic medications, insulin injections, and surgical therapies (bariatric) [27]. However, synthetic medications, such as metformin, can cause adverse effects such as nausea, vomiting, and gastrointestinal disorders [35]. Therefore, the development of affordable and effective diabetes therapies with fewer adverse side effects is a challenge and in high demand [34].

## 3. Functional Beverages

People are now more aware of the nutritional value of their diets [36]. Functional beverages contain bioactive components that come from plants, animals, and microorganisms [37], and they include phenolic compounds, minerals, vitamins, amino acids, peptides, unsaturated fatty acids, and others [38]. To improve the positive consumer perception of functional beverages, researchers have been focusing on enhancing the stability of the active ingredients by encapsulation, emulsion, and high-pressure homogenization procedures.

### 3.1. Definition of Functional Beverages

Currently, there is no universal or legislative definition for the term “functional food”. Nevertheless, according to Regulation (EC) No 1924/2006 of 20 December 2006, functional foods contain nutrients or other substances like vitamins, minerals, amino acids, essential fatty acids, fibre, or plants and herbal extracts that exert a nutritional or physiological effect on consumers [39].

Functional foods are categorically different from nutraceuticals, pharmafood, and nutritional supplements. As a result of their health-promoting properties, which are frequently focused on disease prevention rather than therapeutic effects, they are categorized as food rather than pharmaceutical drugs [40]. In that regard, functional foods and beverages must be consumed in average portions as part of a “normal” diet in order to have beneficial effects [41].

In general, a functional food or beverage can be any of the following: a natural food or beverage; a food or beverage to which a component has been added; a food or beverage from which a component has been removed; a food or beverage whose composition has been altered; a food or beverage in which the bioavailability of an active agent has been modified; or any combination of all of the above [42]. Beverages are a convenient way to consume food and other substances as well as a good way to dissolve active chemicals.

### 3.2. Types of Functional Beverages

According to Corbo et al., functional beverages fall into three categories: sports and energy drinks, dairy-based beverages (including probiotics and minerals-enriched drinks), and beverages made from vegetables and fruits [11]. Fresh milk, fermented milk, and yoghurt drinks are the most popular dairy-based beverages, and they are excellent carriers of probiotics [11]. Sports drinks are flavoured drinks that are meant to be consumed prior to or during exercise to hydrate the body, supply carbohydrates, electrolytes (such as sodium, potassium, calcium, and magnesium), and occasionally, vitamins or other minerals [43]. The main ingredient in energy drinks is typically caffeine, which is intended to improve performance, concentration, and endurance [11]. This last group of beverages imposes a risk on human health due to the high amounts of sugar and acidic additives found in sports drinks, which can cause tooth erosion and decay. Additionally, the high carbohydrate content in these drinks can result in gastrointestinal disorders, with water retention in the intestines, potentially contributing to obesity and damage to the liver. Energy drinks contain guarana and caffeine, which cause lipogenesis: excess fat accelerating its accumulation in the liver, leading to insulin resistance [44]. Particularly for children and adolescents, the high caffeine levels in energy drinks can have several negative effects [45]. Thus, the consumption of this type of drinks must be moderated.

This review will focus on the properties of beverages made from vegetables and fruits, which contain high concentrations of bioactive compounds that could be beneficial in addressing several health issues [17].

### 3.3. Market for Functional Foods and Beverages

**Figure 1:**
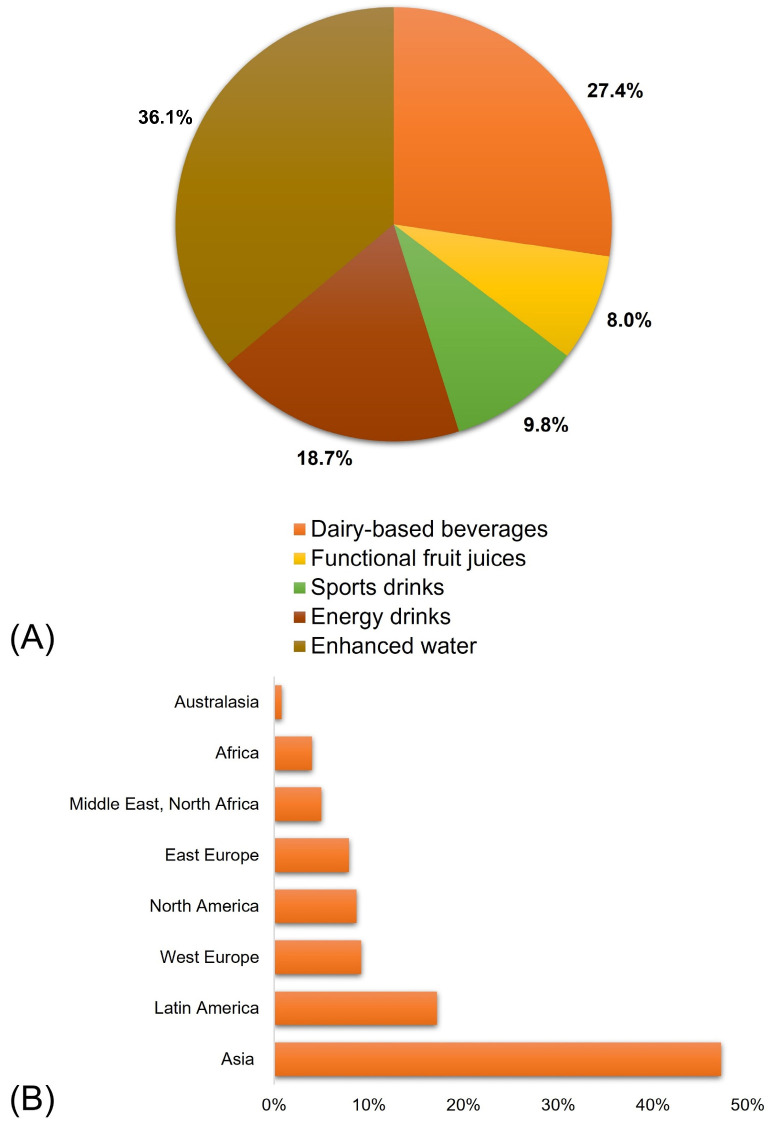
(**A**) Worldwide market of some selected popular beverages in 2019 [48]; (**B**) global beverage consumption forecast (2021) [49].

Functional foods have undeniably emerged as a leading trend in the food industry, despite the lack of clarity on global sales data due to the absence of clear definitions for them [46]. The market for functional foods and beverages was valued at $240.20 billion in 2017, and an increase of $132.84 billion is estimated from 2022 to 2027 [47]. The global market for functional beverages is now dominated by enhanced water, dairy-based beverages, energy drinks, sports drinks, and functional fruit juices (Figure 1A) [48]. The production and consumption of functional beverages have significantly increased as a result of their significant contributions to disease prevention and health promotion, rising urbanization, middle-class population growth, an increase in dual-income households, and growing health concerns [38]. The global functional beverage market was valued at $131.47 billion in 2022, with the Asia-Pacific dominating the worldwide market (Figure 1B) [49]. The need for functional foods varies substantially by country in Europe. The market’s growth has been comparably consistent and profitable, accounting for 16% of global sales. The largest source of income is the United Kingdom, which contributes 20% of total revenues [46].

## 4. The Role of Fruit- and Vegetable-Based Functional Foods and Beverages against Diabetes

Since functional foods are very rich in health-promoting bioactive compounds, particularly antioxidants that actively participate in modulating disease development by inhibiting ROS-mediated reactions in the body, they may help control diseases such as cancer, coronary heart disease, and diabetes [3,50]. Increasing clinical evidence shows that the regular consumption of foods that affect glycaemic control [51], blood pressure regulation [52], activation of antioxidant enzymes [53], and gut microbiota [54] and suppress the excessive production of pro-inflammatory cytokines during diabetes [55] can prevent or delay T2D and its associated complications in high-risk individuals [56]. The use of functional foods as complementary therapy for disease prevention and management has progressively increased over the past few decades, as a strategy to enhance health and psychological well-being. Additionally, this approach has increasingly been adopted by patients aiming to alleviate the adverse effects of traditional medicine and manage the symptoms associated with chronic illnesses [57].

Fruits and vegetables are crucial components of a healthy diet, since they have a low-calorie density and help provide nutrients like vitamins, minerals, dietary fibre, and bioactive substances. They possess attractive colours and flavours and are very hydrating and filling. Their consumption can help replace diets high in salt, sugar, or saturated fats, prevent chronic NCDs like heart disease, cancer, diabetes, and obesity, and address micronutrient deficiencies. At all ages, they promote bodily functioning, development, and physical, mental, and social well-being. In addition to reducing the risk of NCDs [58,59], they can prevent various forms of malnutrition including undernutrition, micronutrient deficiencies, overweightness, and obesity. Along with malnutrition, unhealthy diets are among the top ten global risk factors for disease [60,61].

Li et al. found that an increase in the consumption of fruits and green leafy vegetables, according to previous studies, is linked to a reduced risk of developing T2D [62]. According to Anderson et al., fruits and vegetables have a preventive impact on diabetes because of antioxidants such as polyphenols [63]. The majority of secondary metabolites in plants are phenolic compounds, which are a diverse category of substances that includes simple flavonoids, phenolic acids, complex flavonoids, and coloured anthocyanins [64,65]. According to Survay et al., fruits and vegetables have a hypoglycaemic effect that is attributed to their insulin-like activity [66]. Jayaprakasam et al. and Wedick et al. suggest that this activity may also be due to the increase of insulin secretion by bioactive compounds—anthocyanins and anthocyanidins (insulin secretagogues) [67,68]. The phenolic antioxidants found in berries, in particular, have a significant potential to manage T2D by controlling hyperglycaemia and the microvascular problems associated with cellular oxidative damage as well as macrovascular complications such as hypertension [65].

To date, some research has been conducted on fruit- and vegetable-based functional beverages and their direct or indirect effects on diabetes. This research is primarily focused on the different effects on the metabolism of glucose and insulin, the lipid profile, and the antioxidant properties. In the next sections, we explore this innovative potential of functional beverages as a cutting-edge strategy for diabetes management. Table 1 and table below summarize the findings from 2010. Table 2 outlines the main bioactive compounds of the fruits and vegetables used in the production of each studied functional beverage that are accountable for their biological activity.

### 4.1. In Vitro Studies with Functional Beverages and Diabetes

#### 4.1.1. *α*-Glucosidase and *α*-Amylase Inhibition

According to several authors, functional beverages could represent an innovative strategy for managing diabetes. Table 1 summarises studies indicating that natural functional beverages exhibit an inhibitory effect on *α*-glucosidase and/or *α*-amylase enzymes, which break down polysaccharides into glucose [16]. The inhibition values of *α*-glucosidase and *α*-amylase were reported in the range from 6.12 to 98.6% and 20.03 to 60.14%, respectively [16,69,70]. The ranges for the IC_50_ are 1 to 40.68 and 0.25 to 71.28 mg/mL, respectively [71,72,73]. These results vary depending on the concentration and administered amounts of the studied functional beverage. The small intestine’s *α*-amylase enzyme is crucial in the breakdown of starch into glucose and maltose, consequently increasing the postprandial glucose levels. According to Ujiroghene et al., inhibiting or reducing this enzyme’s ability to digest starch may contribute to the management of diabetes [72]. However, excessive inhibition of *α*-amylase is not advised as it may lead to an accumulation of undigested carbohydrates in the colon, which could promote unfavourable bacterial fermentation and cause flatulence and diarrhoea [74]. *α*-glucosidase is another key enzyme that catalyses the final step in the digestive process of carbohydrates, and its inhibition could similarly delay the digestion of oligosaccharides and disaccharides into monosaccharides, thus reducing glucose absorption and consequently decreasing postprandial hyperglycaemia [75].

According to Badejo et al., the phenolics and flavonoids present in these beverages may be responsible for the inhibitory effect on these enzymes. Polyphenolic compounds may bind covalently to *α*-amylase and modify its activity by forming quinones or lactones that react with nucleophilic groups on the enzyme molecule [70]. Therefore, a viable therapeutic strategy for the treatment of diabetes involves the controlled inhibition of the *α*-amylase and *α*-glucosidase enzymes by the compounds present in these natural functional beverages. The *Prunus* fruit smoothies examined by Nowicka et al. are one of the most active beverages with low IC_50_ values (Table 1). These beverages included sour cherry, peach, apricot and plum fruit, and their bioactive compositions are described in Table 2. The main compounds found in these fruits include chlorogenic acid, catechin, and rutin. Chlorogenic acid has been shown to inhibit *α*-amylase and *α*-glucosidase enzymes by binding to the *α*-amylase-substrate complex and combining with *α*-glucosidase [76]. Catechin’s inhibitory effect on both enzymes has also been demonstrated [77].

The sea buckthorn-based smoothies from Tkacz et al.’s study demonstrated inhibition of pancreatic lipase, primarily due to polymeric procyanidins found in the buckthorn fruit. This inhibition was in addition to that of *α*-amylase and *α*-glucosidase enzymes [16]. Pancreatic lipase is an enzyme that breaks down triglycerides into bioavailable fatty acids and monoglyceride/glycerol molecules. Inhibiting this enzyme contributes to a reduction in energy intake, which can facilitate the control of diabetes [78]. Quercetin and rutin, compounds present in this fruit (Table 2), have been shown to be pancreatic lipase inhibitors [79,80].

#### 4.1.2. Glucose Uptake

To examine the antidiabetic potential of fruit juices, Mahmoud et al. and Zhong et al. tested glucose uptakes after the consumption of *Momordica charantia* (bitter gourd) juice and probiotics-fermented blueberry juice, respectively [73,81]. The glucose uptake assay was used to evaluate the antidiabetic activity of compounds that increase glucose uptake. The consequences of diabetes are caused by high blood glucose levels, which must be reduced to prevent them [82]. The *M. charantia* juice was able to stimulate glucose uptake in the diaphragms of diabetic rats, especially when combined with the administration of insulin. This may be related to the increase of the tissue’s sensitivity to insulin and the potentiation of its action, with charantin being the compound responsible for the diabetic potential of the fruit [81]. Probiotics-fermented blueberry juices also promoted glucose consumption in HepG2 cell lines, highlighting the potential of phenolic compounds to prevent the progression of obesity and hyperglycaemia [73]. A study revealed that the anthocyanin malvidin 3-*O*-galactoside in blueberry improves glucose uptake in HepG2 cells, with malvidin-type anthocyanins exhibiting greater glucose uptake activity compared to delphinidin-type anthocyanins [83]. Blueberry is rich in both types of anthocyanins (Table 2). Castro-Acosta et al. found that apple and blackcurrant polyphenols decrease both sodium-dependent and -independent glucose uptake in Caco-2 cells, which are models for human enterocytes [84]. The apple extract may have inhibited glucose transport in the small intestine since Caco-2 cells are a reliable in vitro model of the human enterocyte. The same extract dose-dependently decreased the total glucose absorption and sodium-independent glucose uptake. This suggests that the control of glucose uptake by polyphenols from natural sources can be a possible approach for the management of blood glucose levels in diabetes. Further research is needed in the development of novel beverages, fruits, and vegetables possessing anti-diabetic properties. Crucially, clinical trials are necessary to validate the above claims. The current in vivo research on this topic will be covered in the following section.

**Table 1:** Reported in vitro assays for anti-diabetic properties of different fruit and vegetable-based functional drinks since 2010.

Beverage	Assays	Results	Reference
Fermented bitter gourd juice	*α*-glucosidase inhibition (measured as glucose production reduction)	↓ glucose production = 14.5–19.2%	[69]
*Prunus* fruit smoothies	*α*-amylase and *α*-glucosidase inhibition	IC_50_ amy ≤ 1.00–8.03 mg/mL IC_50_ gluco = 1.20–6.94 mg/mL	[71]
Bitter gourd fruit juice	Glucose uptake by diaphragms from diabetic rats	Glucose uptake (absence of insulin): ↑ 1.40 mg/g tissue Glucose uptake (presence of insulin): ↑ 4.08 mg/g tissue	[81]
Apple and blackcurrant polyphenol-rich drinks	Glucose uptake by Caco-2 cells	↓ glucose uptake (apple polyphenols) = 46–51% IC_50_ (blackcurrant polyphenols) = 0.51–0.63 mg/mL	[84]
Fermented sprouted quinoa yoghurt beverages	*α*-amylase inhibition	IC_50_ amy (100 µL) = 30.48–39.36 mg/mL IC_50_ amy (200 µL) = 39.44–51.57 mg/mL IC_50_ amy (400 µL) = 50.06–71.28 mg/mL	[72]
Tigernut beverages fortified with extracts of *Vernonia amygdalina* and bitter gourd	*α*-amylase and *α*-glucosidase inhibition	Inhibition amy = 20.59–60.14% Inhibition gluco = 38.82–75.54%	[70]
Probiotics-fermented blueberry juices	*α*-amylase and *α*-glucosidase inhibition Glucose uptake by HepG2 cells	IC_50_ amy = 0.25–2.67 mg/mL IC_50_ gluco = 1–40.68 mg/mL ↑ glucose uptake ≈ 1 mmol/L	[73]
Sea-buckthorn based smoothies	*α*-amylase, *α*-glucosidase and pancreatic lipase inhibition	Inhibition amy = 20.03–49.82% Inhibition gluco = 6.12–98.61% Inhibition lipase = 50.80–96.31%	[16]

Amy = *α*-amylase; gluco = *α*-glucosidase.; IC_50_ = half-maximal inhibitory concentration; ↑ = increase; ↓ = decrease.

**Table 2:** Main bioactive compounds of the fruits and vegetables used in the making of the studied functional beverages.

Fruit/Vegetable	Main Bioactive Compounds	References
Bitter gourd (*Momordica charantia*)	Charantin, polypeptide-P, vicine	[85,86,87]
Sour cherry (*Prunus cerasus*)	Chlorogenic acid, rutin, diadzin, amygdalin, quercetin, naringenin, gallic acid	[88]
Peach (*Prunus persica*)	Protocatechuic, *p*-hydroxybenzoic, *p*-hydroxyphenylacetic, chlorogenic, *p*-coumaric and ferulic acids, catechin	[89]
Apricot (*Prunus armeniaca*)	Catechin, chlorogenic acid, rutin	[90]
Plum fruit (*Prunus* cv. ‘Promis)	n.f.	
Apple (*Malus sylvestris*)	Catechin, chlorogenic acid, epicatechin, hyperoside, quercitrin, phloridzin	[91]
Blackcurrant *	Neochlorogenic acid, chlorogenic acid, epigallocatechin, catechin, epicatechin, myricetin malonyl-glucoside, delphinidin 3-*O*-glucoside, delphinidin 3-*O*-rutinoside, cyanidin 3-*O*-glucoside, cyanidin 3-*O*-rutinoside	[92]
Quinoa *	Hydroxybenzoic acid, vanillic acid, syringic acid, coumaric acid, ferulic acid	[93]
Tigernut (*Cyperus esculentus*)	Oleuropin, pyrogallol, catechin, chlorogenic acid, calicylic acid	[94]
Blueberry (*Vaccinium corymbosum*)	Delphinidin-3-galactoside, malvidin-3-galactoside, malvidin-3-glucoside	[95]
Sea-buckthorn (*Hippophaë rhamnoides*)	Orhamnetin 3-*O*-rutinoside, isorhamnetin 3-*O*-glucoside, isorhamnetin 3-glucoside 7-rhamnoside, isorhamnetin 3-neohesperidoside, quercetin 3-rutinoside, quercetin 3-*O*-glucoside, kaempferol 3-sorphoroside-7-*O*-rhamnoside, isorhamnetin 3-*O*-sorphoroside-7-*O*-rhamnoside, rutin, free isorhamnetin	[96,97,98]
Indian Gooseberry (*Emblica officinalis*)	Myricetin, tannic acid, syringic acid, coumaric acid, caffeic acid, gallic acid	[99]
Noni (*Morinda citrifolia*)	Damnacanthal, morindone, morindin, caproic acid, caprylic acid, xeronine	[100]
Tomato (*Solanum lycopersicum*)	Lycopene, quercetin, kaempferol, naringenin, caffeic acid, lutein	[101]
Pomegranate (*Punica granatum*)	Punicic acid, punicalagin, ellagic acid, gallic acid, oleanolic acid, ursolic acid and uallic acids	[102]
Grapefruit *	Narirutin, naringin, naringenin	[103]
Palm fruit (*Elaeis guineensis*)	Protocatechuic acid, *p*-hydroxybenzoic acid, caffeic acid, *p*-coumaric acid, ferulic acid, 2,4-dimethoxybenzoic acid, cinnamic acid	[104]
Strawberry (*Fragaria* × *ananassa*)	Catechin, pelargonidin, quercetin glucuronides, delphinidin, kaempferol derivatives	[105]
Cranberry (*Vaccinium macrocarpon*)	Peonidin 3-*O*-galactoside, peonidin 3-*O*-arabinoside, cyanidin 3-*O*-galactoside, cyanidin 3-O-arabinoside, myricetin 3-galactoside, quercetin 3-galactoside, quercetin-3-*α*-L-arabinofuranoside, quercetin 3-rhamnoside, ursolic acid, oleanolic acid	[106]
Cowpea (*Vigna Sinensis*)	n.f.	
Açaí (*Euterpe oleracea* Mart.)	Vanillic acid, syringic acid, *p*-hydroxybenzoic acid, protocatechuic acid, ferulic acid, catechin, procyanidin oligomers	[107]
Clementine (*Citrus clementina*)	Narirutin, naringin, (neo)hesperidin	[108]
Gooseberry (*Physalis angulata*)	Cholesteryl acetate, Lupeol acetate, *α*-Tocopherol	[108]
*Bengkuang* *	n.f.	
Pigeon pea (*Cajanus cajan*)	Quercetin, quercetin 3-*O*-glucoside, quercetin 3-*O*-methylether, isoprenylated-genistein, cajanol, cajanin	[109]

n.f. = not found; * = species not specified.

### 4.2. In Vivo Studies with Functional Beverages and Diabetes

Some in vivo research has been conducted to explore the anti-diabetic properties of functional beverages (Table 3). Rats are a suitable animal model used to understand the mechanisms of diabetes, with streptozotocin and alloxan being the most common chemical agents applied for the induction of diabetes in rats [110]. Several studies have examined the efficacy of different functional beverages in preventing diabetes in streptozotocin- or alloxan-induced diabetic rats. Other studies have employed the induction of obesity in rats, via the administration of a high-fat diet, to assess the impact of vegetable and fruits on the development of diabetes in obese mice. Effects have been demonstrated on body weight, glucose and/or insulin metabolism, lipid profile, and antioxidant status.

#### 4.2.1. Effects on Body Weight

Obesity, caused by the excessive accumulation of adipose tissue in the body, is a highly prevalent metabolic disorder, and its progression leads to T2D and associated health issues [111]. As shown by several authors [112,113,114,115], different functional beverages have helped obese rat models lose body weight. In a study by Seo et al., reduction in body weight, resulting from the consumption of a tomato and vinegar beverage, was attributed to increased fatty acid oxidation rather than inhibition of lipid biosynthesis [113]. One of the main compounds of tomato is quercetin (Table 2) that has been previously associated with fatty acid oxidation resulting from lipophagy in hepatocytes, according to a study of Fukaya et al. [116]. Another study showed that the *Emblica officinalis* fruit juice reduced the body weight of mice. The effect of gallic acid from *E. officinalis* was also tested in the same study and showed the same results. According to the authors, gallic acid is able to activate the PPAR-*α* (peroxisome proliferator-activated receptor) and lipid metabolism of the high fat-induced obese rats [115]. PPAR-*α* is a key regulator of energy homeostasis and controls the expression of genes involved in fatty acid *β*-oxidation [117].

On the other hand, weight loss from the degeneration of adipocytes and muscular tissues to compensate for the body’s energy loss, which is caused by frequent urination and excessive glucose being transferred from glycogen, is an important aspect of managing diabetes. A few studies have indicated the control of body weight loss in diabetic rats through the consumption of different fruit/vegetable drinks [118,119,120,121]. However, the specific mechanisms underlying this effect have not yet been discussed.

#### 4.2.2. Effects on Glucose and Insulin Metabolism

Diabetes is characterized by increased fasting blood glucose, hyperinsulinemia, and insulin resistance [122]. Diabetes is diagnosed when the fasting plasma glucose level exceeds 126 mg/dL, or the casual plasma glucose is >200 mg/dl [123]. In these investigations, the ability of fruit juices to lower hyperglycaemia was frequently reported as causing a reduction in fasting blood, plasma, or serum glucose levels. For instance, Ariviani et al. showed the hypoglycaemic effect of a pigeon pea beverage on diabetic rats due to the antioxidant compounds that possess the ability to scavenge free radicals, which improves insulin secretion and as a result, decreases blood glucose levels [118]. Pigeon pea’s main bioactive compounds include quercetin, quercetin 3-*O*-glucoside, and quercetin 3-*O*-methylether (Table 2). The oral administration of quercetin in doses ranging from 15 to 100 mg/kg body weight for periods spanning 14 to 70 days demonstrated a reduction in blood glucose levels in diabetic rat models by increasing serum insulin levels, thus promoting the release of insulin and regenerating pancreatic islets [124]. According to the literature, this flavonoid’s hypoglycaemic mechanisms may involve the following: promotion of the proliferation of pancreatic *β*-cells, their protection against oxidative damage, and the increase of insulin secretion from these cells [125,126]; enhancement of glucose uptake in organs and tissues [127]; improvement of insulin resistance [128]; and reduction of intestinal glucose absorption by the inhibition of the *α*-glucosidase enzyme [129].

The antidiabetic effect of Noni (*Morinda citrifolia*)’s fruit juice was attributed to the regulation of the FoxO1 mRNA expression. The phosphorylation of the FoxO1 transcription factor inhibits gluconeogenic enzymes, improving glucose metabolism [112]. This fruit contains damnacanthol (Table 2), which has been found to have hypoglycaemic effects [130].

Insulin resistance is defined by compensatory hyperinsulinemia due to a decreased sensitivity of target tissues, such as skeletal muscles, the liver, and adipose tissue, to insulin [131]. In an attempt to counteract hyperglycaemia, increased insulin production results in hyperinsulinemia [115]. In diabetics, the body loses the ability to produce insulin, which is caused by pancreatic *β*-cell apoptosis or insulin resistance [132]. According to Mahmoud et al.’s hypothesis, *M. charantia* in fruit juice has the ability to decrease blood glucose in diabetic rats by stimulating the surviving *β*-cells to release more insulin [81]. *M. charantia* is well known for its antidiabetic properties, mainly because it contains a compound named charantin (Table 2), which has blood glucose-lowering properties similar to insulin [133]. A tomato and vinegar beverage improved postprandial glucose levels with decreased plasma insulin levels, demonstrating the reduction of insulin resistance [113]. This was attributed to the reduction of free fatty acid concentration in obese rats, which induces hepatic fat accumulation, leading to a decrease in insulin sensitivity and the production of glucose. In this study, an increase in the activity of the enzyme glucokinase (GCK) was observed. Reduced GCK activity has been associated with poor insulin production by pancreatic *β*-cells and glucose tolerance [112]. The main bioactive compound of tomato is lycopene (Table 2), which has been found to increase the activity of pancreatic GCK in diabetic rat models [134]. This is one of the possible routes that can be used to target hyperglycaemia in diabetes.

Variya et al.’s study on high fructose-induced diabetic rats demonstrated that gallic acid from *E. officinalis* decreased insulin resistance by activating Akt. This is a protein that has a role in the transcriptional activation of PPAR-*γ*. which is a receptor involved in the expression of the *GLUT-4* (glucose transporter type 4) gene [115]. *GLUT-4* mediates the circulation, glucose reduction, and body homeostasis, and its inappropriate translocation is caused by damaged insulin responders/signalling [135,136]. This juice was able to decrease arterial blood pressure that had been increased by fructose. According to Iwansyah et al., drinking fruit juice from *P. angulata* also increases the expression of the *GLUT-4* gene in diabetic rats. This resulted in the increased absorption of blood glucose into cells, thus decreasing glucose levels [121].

Grapefruit juice has been shown to have an antiglycaemic effect as strong as that of metformin medication [114]. This juice significantly lowered fasting serum insulin in diabetic rats. Naringin in the juice, one of the main compounds of grapes (Table 2), had a lowering effect on blood glucose levels and insulin resistance. This compound’s antidiabetic effect has been attributed to the enhancement of insulin sensitivity by the activation of AMPK (AMP-activated protein kinase), which is involved in insulin signalling [137].

Palm fruit juice has anti-hyperglycaemic effects on diabetic rats, which is explained by a decrease in insulin resistance, a reduction in glucose absorption, or an increase in insulin secretion. With a low intake of palm fruit phenolics, the rats showed low insulin levels, while with higher amounts, the plasma insulin increased, demonstrating a possible increase in insulin secretion [119]. A later study tried to investigate the molecular mechanisms of the anti-diabetic effects of palm fruit juice [138]. The treatment of T2D-induced rats with the juice led to the up-regulation of 71 hepatic genes, including apolipoproteins related to high-density lipoproteins and genes involved in hepatic detoxifications. The treatment down-regulated 108 genes related to insulin signalling and fibrosis. With these results, the authors concluded that the mechanism of action of palm fruit phenolics is not only related to increases in insulin secretion.

Numerous secondary disorders, such as obesity, cardiovascular problems, hypertension, hypertriglyceridemia, and atherosclerosis, are mostly attributed to insulin resistance [139]. Treatments that can boost insulin sensitivity and reduce endogenous insulin levels are suitable approaches to manage diabetes and its metabolic complications [140].

#### 4.2.3. Effects on the Lipid Profile

In diabetes mellitus, hyperglycaemia and dyslipidaemia coexist. Insulin resistance leads to a more atherogenic lipid profile [141], and diabetics can benefit from medication that also regulates abnormal lipid levels [142]. The high consumption of fruits and vegetables has been linked to decreased plasma lipid levels [143]. Some studies on fruit/vegetable juices have demonstrated improvements in the lipid profiles of diabetic rats. The beverage administration of pigeon pea was able to significantly lower the cholesterol levels of rats with hypercholesteremia. The authors of the study attribute this to dietary fibre, which regulates the HMG-CoA reductase expression that is responsible for the production of cholesterol, and to the antioxidant capacity of the beverage with antioxidants inhibiting the oxidation of LDL cholesterol and suppressing its uptake in macrophages [118]. The gallic acid from *E. officinalis* fruit juice led to decreases in cholesterol and triglycerides in fructose-induced diabetic rats [115]. Methods to modify blood lipids are necessary to lessen the risk of problems caused by and the progression of diabetes, since high levels of the total cholesterol and triglycerides may lead to cardiovascular complications.

#### 4.2.4. Antioxidant Status

Diabetes and its complications are thought to be caused by oxidative stress, which can be a mediator of insulin resistance and its progression to glucose intolerance [144]. Malondialdehyde (MDA) is a biomarker for oxidative stress in diabetes mellitus and is linked to lipid peroxidation. A high plasma level in this marker indicates low antioxidant status [118]. The *M. charantia* fruit juices used by Mahmoud et al. and Gao et al. were able to mitigate oxidative stress, as shown by the reduction of MDA levels [81,120]. Ariviani et al. found that administering a pigeon pea beverage to diabetic-hypercholesterolemic rats also reduced their MDA levels [118]. *M. charantia* juice also led to an increase in the TAOC (total antioxidant capacity) and pancreatic GSH levels (pancreatic reduced glutathione) [81]. They attribute this effect to the augmented synthesis of GSH and other antioxidant enzymes, a reduction of oxidative stress and consequent reduction in the degradation of those enzymes, or a combination of both.

Several different mechanisms have been proposed regarding the anti-diabetic activity of natural juices, and they are still being studied. The next step is to perform clinical trials to evaluate their effect on humans.

### 4.3. Clinical Trials with Functional Beverages and Diabetes

A few clinical studies have been performed to validate the antidiabetic effect of functional beverages on patients with diabetes. Banihani et al. reported a decrease in fasting serum glucose and insulin resistance in patients with T2D, 3 h after the consumption of fresh pomegranate juice, at 1.5 mL/kg of body weight. In addition, they reported an increase in the *β*-cell function [145]. Pomegranate is rich in compounds such as punicid acid and punicalagin (Table 2), which have been reported to stimulate PPARs [146] and insulin secretion [147], respectively. In a clinical study performed by Devaki and Premavalli, 6 months of daily consumption of 45 mL of an *M. charantia*-fermented beverage (equivalent to a dose of 18 mg of phenols, 129 mg of quinine, and small quantities of five different vitamins every day) in diabetic subjects led to an improvement of symptoms. There was a significant reduction of fasting blood glucose, postprandial blood glucose, and HbA1c (glycated haemoglobin) levels [148]. HbA1c levels reflect the average blood glucose concentration over the past few weeks [5]. The blood lipid profile remained the same. The authors claim that the effect of this drink is due to a lectin with activity similar to insulin contributing to its hypoglycaemic effect. It also contains polypeptide-P (Table 2), which is an insulin-like substance that decreases blood sugar levels.

A clinical trial involving the daily consumption of a beverage enriched with 333 mg of polyphenols from cranberry and strawberry for 6 weeks on 116 insulin-resistant individuals revealed an improvement in insulin sensitivity. However, the beverage did not affect the total LDL and HDL cholesterol or triglycerides or the markers for oxidative stress and inflammation (pro-inflammatory cytokines, C-reactive protein, HMW adiponectin, PAI-1, and oxidised-LDL, RANTES, or total antioxidant capacity of plasma) [149]. According to the author’s results and research, doses of polyphenols lower than 800 mg have metabolic benefits. Kim et al. tested the modulation of the lipid and glucose metabolism and of oxidative stress and inflammation by a beverage made with açaí, which is rich in anthocyanins like cyanidin 3-*O*-rutinoside and cyanidin 3-*O*-glucoside. Here, 37 individuals with metabolic syndrome were randomized and drank 325 mL of the beverage with 1.139 mg/L gallic acid equivalents of total polyphenolics (or a placebo control) twice a day for 12 weeks. At the end of the study, the plasma level of interferon-gamma (IFN-*γ*) and urinary level of 8-isoprostane (inflammatory response and oxidative stress biomarkers, respectively) were significantly decreased, which contribute to reducing the risk of developing chronic diseases. However, the glucose and lipid metabolism biomarkers were not affected [150]. According to a case report by Aktan et al., the daily consumption of *Vaccinium corymbosum* juice for 2 years by a 75-year-old pre-diabetic patient induced profound hypoglycaemia. The serum glucose values were at the level of 30 mg/dl after the episode, and the patient had drunk up to 500 mL of the juice 1–2 h prior. After discontinuing the consumption of the beverage for 6 months, the levels increased to 105 mg/dl [151]. This suggests the important role of *V. corymbosum* juice in lowering serum glucose levels. This fruit (blueberry) is rich in anthocyanins such as delphinidin 3-galactoside, malvidin-3-galactoside, and malvidin 3-glucoside (Table 2). Hasniyati et al. reported that functional yogurt containing *bengkuang* was able to decrease the MDA levels of T2D patients, after 2 weeks of daily consumption by a group of 46 people, but had no impact on fasting blood glucose levels [152]. Lastly, drinks rich in apple and blackcurrant polyphenols had a diabetes-preventing effect, by lowering postprandial plasma glucose levels, C-peptide, GIP, and insulin in 25 healthy men and women, 30 min after the daily dose of 1200 mg apple polyphenols or 600 mg apple polyphenols + 600 mg blackcurrant anthocyanins drinks. The triglyceride levels stayed the same [84].

As of now, enough clinical trials have not been conducted to determine the specific dose of a compound necessary to induce a specific anti-diabetic effect. Moreover, complex polyphenol combinations found in fruit extracts might be responsible for these effects, which are potentially due to additive or synergistic actions [84]. Due to the complexity of these foods, their effects might not be solely attributed to one or two specific compounds, and the outcomes may differ from one beverage to another even if they contain the same fruit.

Regarding safety, the consumption of natural polyphenols is relatively high in a typical human diet, and they have an excellent safety profile [153]. For example, a study demonstrated that a daily dose of 320 mg of anthocyanins has positive effects on dyslipidaemia and insulin resistance in diabetic patients [154]. Polyphenols from blueberries have been found to be safe for consumption up to 1000 mg/kg body weight in rats for 90 days [155]. Currently, there is little evidence to suggest toxicity from polyphenols at higher dosages, since most of the polyphenol research has been focused on determining the lowest amount required to show a positive health impact. Although the dose of individual polyphenols can be investigated using traditional toxicological or pharmacological models, alternative methods are needed to study the complex mixes that consumers are exposed to [156].

**Table 3:** Reported in vivo assays for anti-diabetic properties of different fruit- and vegetable-based functional drinks since 2010.

Beverage	Administration	Relevant Results	Reference
*Emblica officinalis* fruit juice	1 mL/kg, daily, 8 weeks in STZ-DR	↓ serum glucose, FBG, TAG, TC, VLDL-C ↑ serum insulin, FBI, HDL-C, LDL-C	[157]
Fermented noni fruit juice	0.0015 mL/kg, 2 × day, 12 weeks in HFD-OR	↓ body weight, FBG, insulin resistance ↑ insulin, glucose tolerance	[112]
Processed tomato-vinegar beverage	14 mL/kg, daily, 6 weeks in HFD-OR	↓ TAG, body weight, insulin resistance ↑ glucose tolerance, HDL-C, GCK activity	[113]
Fresh pomegranate juice	1.5 mL/kg, once, in T2D patients	↑ *β*-cell function ↓ FPG, insulin resistance	[145]
Bitter gourd fermented beverage	45 mL, daily, for 1 and 6 months in diabetic patients	↓ FBG and PPBS (1 month) ↓ FBG and PPBS, = blood lipid profile, ↑ HbA1c (6 months)	[148]
Grapefruit sweetened juices	2–3 mL, daily, for 2 weeks in HFD-OR	↓ body weight, FBG, FSI, liver TAG	[114]
*Vaccinium corymbosum* infusion	Cup of juice, daily, for 2 years, in a pre-diabetic	↓ serum glucose, HbA1c, insulin resistance	[151]
Palm fruit juice	170–720 mg GAE/kg, daily, for 4–36 weeks in CS-DR	↓ blood glucose, TAG, TC, liver lipids =body weight	[119]
Palm fruit juice	HC diet + 5400 mg GAE/kg, 4 weeks in DR	↓ blood glucose, ↓ body weight, TAG	[138]
Apple and blackcurrant polyphenol-rich drinks	200 g, once, in healthy patients	↓ PPG, insulin, C-peptide, GIP =TAG	[84]
Bitter gourd fruit juice	10 mL/kg, 14 days before diabetes and 21 days after, in STZ-DR	↓ serum glucose, insulin resistance, serum TC, TAG, pancreatic MDA ↑ serum insulin, *β*-cell function, HDL-C, TAOC, pancreatic GSH	[81]
Strawberry and cranberry polyphenols beverage	333 mg polyphenols, daily, for 6 weeks, in insulin-resistant patients	↓ insulin resistance =TC, LDL-cholesterol, HDL-cholesterol, TAG	[149]
Cowpea juice, tomato juice and green apple juices combined	Combinations, daily, for 28 days in ALL-DR	↓ FBG	[35]
Pigeon pea beverage dilluted in water	2700 mg/kg, daily, for 2 weeks in DHR	↓ plasma glucose, TC, MDA =body weight	[118]
Açaí beverage	2 × 325 mL, daily, for 12 weeks, in patients with metabolic syndrome	↓ IFN-*γ* plasma level, 8-isoprostane	[150]
Fermented bitter gourd juice	10 mL/kg, daily, for 4 weeks in STZ-DR	↓ body weight loss, blood glucose, FBG, serum insulin, insulin resistance, TC, LDL-C, TAG, MDA ↑ HDL-C	[120]
*Emblica officinalis* fruit juice	2 mL/kg, daily, for 42 days in DR; for 4 weeks in HF-DR	↓ body weight, FBG, insulin resistance, HbA1c, TAG, blood pressure; TC =HDL-C	[115]
Citrus concentrate enriched with b-cryptoxanthin, hesperidin and pectin	2 mL, daily, for 8 weeks in HF-PDR	↑ glucose tolerance ↓ plasma glucose, plasma insulin, TAG, LDL-C, VLDL-C, blood pressure =TC, HDL-C	[158]
*Physalis angulata* fruit juice	1 and 2 mL/kg, daily, for 2 weeks in STZ-DR	↓ FBG =body weight	[121]
Yogurt bengkuang tape ketan hitam	200 mL, daily, for 2 weeks in T2D patients	=FBG ↓ plasma MDA	[152]

STZ-DR = streptozotocin-induced diabetic rats; ALL = alloxan-induced diabetic rats; HFD-OR = high-fat diet-induced obese rats; CS-DR = carbohydrate-sensitive diabetic rats; HF-PDR = high-fructose diet pre-diabetic rats; T2D = type 2 Diabetes; PD = pre-diabetic; GAE = gallic acid equivalents; FBG = fasting blood glucose; FPG = fasting plasma glucose; PPG = postprandial plasma glucose; FSI = fasting serum insulin; TAG = triglycerides; DHR = diabetic-hypercholesterolemia rats; IFN = interferon-gamma; MDA = malondialdehyde; GIP = Gastric inhibitory polypeptide; AI = atherogenicity; TAOC = total antioxidant capacity; GSH = reduced glutathione; TC = total cholesterol; HDL-C = high-density lipoprotein cholesterol; LDL-C = low-density lipoprotein cholesterol; VLDL-C = very low-density lipoprotein cholesterol; GCK = glucokinase; G6P = glucose-6-phosphatase; HMG-CoA = HMG-CoA reductase; ↑ = increase; ↓ = decrease; = means maintenance of levels.

An interesting aspect of this research is the positive effects observed in certain fermented beverages that were studied. Research has indicated a protective effect of alcoholic beverages against diabetes, particularly with light to moderate consumption [159]. Conigrave and Rimm suggested that the consumption of a small amount of alcohol might even be beneficial in managing cardiac complications of diabetes, as long as it is done in low doses to prevent hypoglycaemia or poor glycaemic control [160]. This highlights the potential of functional beverages to exert beneficial health effects even if they contain alcohol in their composition and provided the consumption levels are controlled.

The carbohydrate content of different fruit and vegetable juices may significantly impact the effects of their consumption. Fruit juice composition varies depending on the species or variety of fruit, maturity, and the environmental and climatic factors of the growing season [161]. Fruit juices with sorbitol and a fructose-to-glucose ratio greater than one are more likely to result in carbohydrate malabsorption, which can induce diarrhoea and stomach pain [162]. For example, white grape and orange juices have an almost equal amount of glucose and fructose and do not contain sorbitol. In pear and apple juices, there is a higher concentration of fructose than glucose, and they contain sorbitol. The carbohydrate composition of the first-mentioned juices favours carbohydrate absorption [162]. This is an important aspect to consider when selecting the type of foods used to produce functional beverages directed at the control of diabetes.

Despite the interesting results from our study, we need to consider the disadvantages of consuming juices instead of whole fruits and vegetables. Unlike fresh fruit, fruit juices are not a good source of fibres, are less satiating, and usually have high sugar content [163]. Therefore, their consumption should not substitute that of fresh fruit and vegetables but should be used as an extra means of ensuring a healthy and balanced diet [7]. Some other limitations include the elevated price of functional beverages, organoleptic issues, which may require the addition of sugar or other sweeteners, and the reduced shelf life of natural beverages. The safety of the consumption of these types of foods must be guaranteed with the use of preservation methods that increase the stability of their shelf life [46].

## 5. Conclusions and Future Perspectives

Diabetes mellitus is a common metabolic disorder with numerous complications, and its incidence has been on the rise globally. This is largely due to lifestyle trends, particularly poor dietary habits and the lack of exercise that contributes to the obesity epidemic overall. The current options for the treatment of diabetes have undesired side effects. More natural and safer alternatives are becoming popular within the community for the control of this and other diseases.

**Figure 2:**
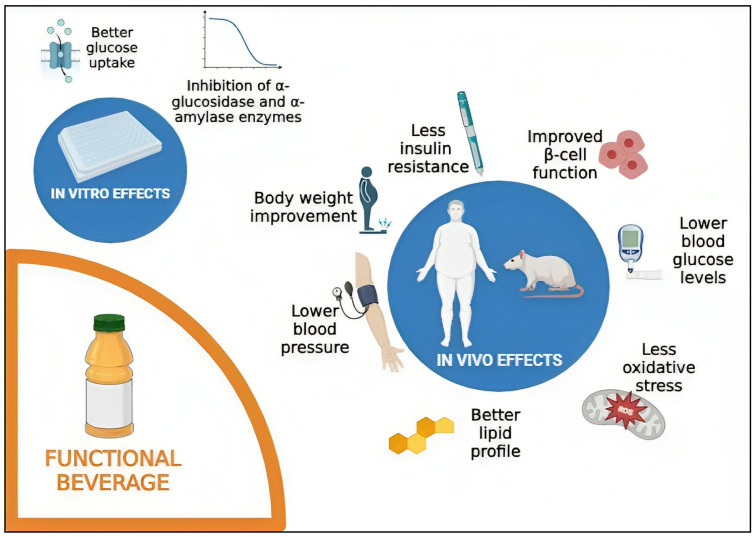
In vitro and in vivo diabetic effects of natural functional beverages. (Created in BioRender.com).

Fruit and vegetable consumption is beneficial for health and highly advised by health organizations. Beverages like juices and smoothies are an easy and pleasant way to include these foods in our diet, and they contain bioactive compounds, mainly polyphenols, that enhance health properties. Therefore, this review has been performed on the effects of fruit- and vegetable-based natural functional beverages on the management of diabetes. Several natural juices have been tested for anti-diabetic properties, both in vitro and in vivo. From the in vitro perspective, they have shown properties such as the ability to inhibit *α*-glucosidase and *α*-amylase enzymes and improve the glucose uptake by different organs (Figure 2). In vivo studies have evidenced many interesting anti-diabetic effects such as the reduction of blood glucose, increase of insulin tolerance, improvement of lipid profiles, control of obesity, and reduction of oxidative stress (Figure 2). This highlights the potential of natural beverages, including those that are alcoholic, as novel anti-diabetic agents. To address these concerns, it could be valuable to develop and test new beverages centred around foods that are rich in bioactive compounds with anti-diabetic properties and introduce them into the diets of diabetics or even obese people who are at risk for the disease. However, the safety of the regular consumption of these products needs to be assessed and well-defined beforehand. There is a need to perform a toxicity evaluation for individual bioactive compounds and understand safe dosage levels before incorporating them into human diets. Afterward, further clinical trials are essential to test the actual effects of these compounds on humans. Additionally, understanding the long-term implications of consuming these functional foods is crucial.

Another interesting approach would be to evaluate the use of these functional beverages as a complement to existing diabetes treatments with the need to assess possible synergistic (or antagonistic) effects between other medications and these foods. From a circular economy perspective, making beverages from fruits and vegetables that are too unattractive to sell and are typically discarded would be a smart move to reduce food waste while also increasing cost efficiency.

## Data Availability

No new data were created or analysed in this study. Data sharing is not applicable to this article.

## References

[1] Gayathry K.S., John J.A. (2021). Functional Beverages: Special Focus on Anti-Diabetic Potential. J. Food Process. Preserv..

[2] World Health Organization Noncommunicable Diseases. https://www.who.int/en/news-room/fact-sheets/detail/noncommunicable-diseases.

[3] Olaiya C.O., Soetan K.O., Esan A.M. (2016). The Role of Nutraceuticals, Functional Foods and Value Added Food Products in the Prevention and Treatment of Chronic Diseases. Afr. J. Food Sci..

[4] World Health Organization (2019). WHO Global Report on Traditional and Complementary Medicine 2019.

[5] World Health Organization (2016). Global Report on Diabetes.

[6] Ong K.L., Stafford L.K., McLaughlin S.A., Boyko E.J., Vollset S.E., Smith A.E., Dalton B.E., Duprey J., Cruz J.A., Hagins H. (2023). Global, Regional, and National Burden of Diabetes from 1990 to 2021, with Projections of Prevalence to 2050: A Systematic Analysis for the Global Burden of Disease Study 2021. Lancet.

[7] Caswell H. (2009). The Role of Fruit Juice in the Diet: An Overview. Nutr. Bull..

[8] Beam A., Clinger E., Hao L. (2021). Effect of Diet and Dietary Components on the Composition of the Gut Microbiota. Nutrients.

[9] Kramer H. (2019). Diet and Chronic Kidney Disease. Adv. Nutr..

[10] Ojo O. (2019). Nutrition and Chronic Conditions. Nutrients.

[11] Corbo M.R., Bevilacqua A., Petruzzi L., Casanova F.P., Sinigaglia M. (2014). Functional Beverages: The Emerging Side of Functional Foods Commercial Trends, Research, and Health Implications. Food Sci. Food Saf..

[12] Selcuk M.Y., Aygen B., Dogukan A., Tuzcu Z., Akdemir F., Komorowski J.R., Atalay M., Sahin K. (2012). Chromium Picolinate and Chromium Histidinate Protects against Renal Dysfunction by Modulation of NF-B Pathway in High-Fat Diet Fed and Streptozotocin-Induced Diabetic Rats. Nutr. Metab..

[13] Rafighi Z., Shiva A., Arab S., Mohd Yousof R. (2013). Association of Dietary Vitamin C and E Intake and Antioxidant Enzymes in Type 2 Diabetes Mellitus Patients. Glob. J. Health Sci..

[14] Shoji T., Yamada M., Miura T., Nagashima K., Ogura K., Inagaki N., Maeda-Yamamoto M. (2017). Chronic Administration of Apple Polyphenols Ameliorates Hyperglycaemia in High-Normal and Borderline Subjects: A Randomised, Placebo-Controlled Trial. Diabetes Res. Clin. Pract..

[15] Kim M. (2005). High-Methoxyl Pectin Has Greater Enhancing Effect on Glucose Uptake in Intestinal Perfused Rats. Nutrition.

[16] Tkacz K., Wojdyło A., Turkiewicz I.P., Nowicka P. (2021). Anti-Diabetic, Anti-Cholinesterase, and Antioxidant Potential, Chemical Composition and Sensory Evaluation of Novel Sea Buckthorn-Based Smoothies. Food Chem..

[17] Dey G., Sireswar S. (2021). Tailoring Functional Beverages from Fruits and Vegetables for Specific Disease Conditions-Are We There Yet?. Crit. Rev. Food Sci. Nutr..

[18] Liu J., Ren Z.H., Qiang H., Wu J., Shen M., Zhang L., Lyu J. (2020). Trends in the Incidence of Diabetes Mellitus: Results from the Global Burden of Disease Study 2017 and Implications for Diabetes Mellitus Prevention. BMC Public Health.

[19] United Nations (2011). Political Declaration of the High-Level Meeting of the General Assemblyon the Prevention and Control of Noncommunicable Diseases.

[20] International Diabetes Federation (2013). IDF Diabetes Atlas.

[21] Wootton-Beard P.C., Ryan L. (2011). Improving Public Health?: The Role of Antioxidant-Rich Fruit and Vegetable Beverages. Food Res. Int..

[22] Zhou B., Lu Y., Hajifathalian K., Bentham J., Di Cesare M., Danaei G., Bixby H., Cowan M.J., Ali M.K., Taddei C. (2016). Worldwide Trends in Diabetes Since 1980: A Pooled Analysis of 751 Population-Based Studies with 4.4 Million Participants. Lancet.

[23] Atkinson M.A., Eisenbarth G.S., Michels A.W. (2014). Type 1 Diabetes. Lancet.

[24] Deshpande A.D., Harris-Hayes M., Schootman M. (2008). Epidemiology of Diabetes and Diabetes-Related Complications. Phys. Ther..

[25] Patterson C.C., Harjutsalo V., Rosenbauer J., Neu A., Cinek O., Skrivarhaug T., Rami-Merhar B., Soltesz G., Svensson J., Parslow R.C. (2019). Trends and Cyclical Variation in the Incidence of Childhood Type 1 Diabetes in 26 European Centres in the 25 Year Period 1989–2013: A Multicentre Prospective Registration Study. Diabetologia.

[26] Patterson C.C., Dahlquist G.G., Gyürüs E., Green A., Soltész G., Group E.S. (2009). Incidence Trends for Childhood Type 1 Diabetes in Europe during 1989–2003 and Predicted New Cases 2005-20: A Multicentre Prospective Registration Study. Lancet.

[27] Venkatakrishnan K., Chiu H.F., Wang C.K. (2019). Popular Functional Foods and Herbs for the Management of tyPe-2-Diabetes Mellitus: A Comprehensive Review with Special Reference to Clinical Trials and Its Proposed Mechanism. J. Funct. Foods.

[28] Li P., Tang Y., Liu L., Wang D., Zhang L., Piao C. (2019). Therapeutic Potential of Buckwheat Hull Flavonoids in Db/Db Mice, a Model of Type 2 Diabetes. J. Funct. Foods.

[29] Wu Y., Ding Y., Tanaka Y., Zhang W. (2014). Risk Factors Contributing to Type 2 Diabetes and Recent Advances in the Treatment and Prevention. Int. J. Med. Sci..

[30] Tinajero M.G., Malik V.S. (2021). An Update on the Epidemiology of Type 2 Diabetes: A Global Perspective. Endocrinol. Metab. Clin. N. Am..

[31] World Health Organization Diabetes. https://www.who.int/europe/health-topics/diabetes#tab=tab_1.

[32] Centers for Disease Control and Prevention Diabetes Tests. https://www.cdc.gov/diabetes/basics/getting-tested.html.

[33] (2022). Saad Masood Butt Management and Treatment of Type 2 Diabetes. Int. J. Comput. Inf. Manuf..

[34] Koh S.P., Maarof S., Sew Y.S., Sabidi S., Abdullah R., Mohd Danial A., Nur Diyana A., Mustaffa R. (2020). Fermented Jackfruit Leaf Beverage Offers New Affordable and Effective Diabetes Therapy. Food Res..

[35] Naim A., Anisa L., Marjoni R. (2018). Antidiabetes Effects—Combination of Cowpea Juice (*Vigna sinensis* L.), Tomato Juice (*Solanum lycopersicum* L.), and Green Apple Juice (*Malus sylvestris* Mill.) in White Male Mice. Int. J. Green Pharm..

[36] Gupta A., Sanwal N., Bareen M.A., Barua S., Sharma N., Joshua Olatunji O., Prakash Nirmal N., Sahu J.K. (2023). Trends in Functional Beverages: Functional Ingredients, Processing Technologies, Stability, Health Benefits, and Consumer Perspective. Food Res. Int..

[37] Manousi N., Sarakatsianos I., Samanidou V. (2019). Extraction Techniques of Phenolic Compounds and Other Bioactive Compounds From Medicinal and Aromatic Plants. Engineering Tools in the Beverage Industry.

[38] Raman M., Ambalam P., Doble M. (2019). Probiotics, Prebiotics, and Fibers in Nutritive and Functional Beverages. Nutrients in Beverages.

[39] European Parliament and the Council of the European Union (2006). Regulation (EC) 1924/2006 on Nutrition and Health Claims Made on Foods.

[40] Gonçalves A.C., Nunes A.R., Flores-Félix J.D., Alves G., Silva L.R. (2022). Cherries and Blueberries-Based Beverages: Functional Foods with Antidiabetic and Immune Booster Properties. Molecules.

[41] Cong L., Bremer P., Mirosa M. (2020). Functional Beverages in Selected Countries of Asia Pacific Region: A Review. Beverages.

[42] Henry C.J. (2010). Functional Foods. Eur. J. Clin. Nutr..

[43] Heckman M.A., Sherry K., de Mejia E.G. (2010). Energy Drinks: An Assessment of Their Market Size, Consumer Demographics, Ingredient Profile, Functionality, and Regulations in the United States. Compr. Rev. Food Sci. Food Saf..

[44] Sugajski M., Buszewska-Forajta M., Buszewski B. (2023). Functional Beverages in the 21st Century. Beverages.

[45] Sikalidis A.K., Kelleher A.H., Maykish A., Kristo A.S. (2020). Non-Alcoholic Beverages, Old and Novel, and Their Potential Effects on Human Health, with a Focus on Hydration and Cardiometabolic Health. Medicina.

[46] Grumezescu A.M., Holban A.M., Grumezescu A.M., Holban A.M. (2019). Functional and Medicinal Beverages Volume 11: The Science of Beverages.

[47] Technavio Functional Foods and Beverages Market by Product, Distribution Channel, and Geography—Forecast and Analysis 2023–2027. https://www.technavio.com/report/functional-foods-and-beverages-market-industry-analysis.

[48] DataIntelo (2019). Global Functional Beverages Market by Types (Dairy Based Beverages, Enhanced Water, Energy Drinks, Sports Drinks, Functional Fruit Juices, and Others), Distribution Channels (Store Based, Online, Supermarkets & Hypermarkets, and Convenience Stores), and Regions (Asia Pacific, Europe, North America, Middle East & Africa, and Latin America)—Global Industry Analysis, Growth, Share, Size, Trends, and Forecast from 2022 to 2030.

[49] Arthur R. (2016). Unprecedented Growth for Asia Beverage Market.

[50] Ashaolu T.J., Adeyeye S.A.O. (2022). African Functional Foods and Beverages: A Review. J. Culin. Sci. Technol..

[51] Hegde S.V., Adhikari P., Nandini M., D’Souza V. (2013). Effect of Daily Supplementation of Fruits on Oxidative Stress Indices and Glycaemic Status in Type 2 Diabetes Mellitus. Complement. Ther. Clin. Pract..

[52] Shidfar F., Froghifar N., Vafa M., Rajab A., Hosseini S., Shidfar S., Gohari M. (2011). The Effects of Tomato Consumption on Serum Glucose, Apolipoprotein B, Apolipoprotein A-I, Homocysteine and Blood Pressure in Type 2 Diabetic Patients. Int. J. Food Sci. Nutr..

[53] Potter A.S., Foroudi S., Stamatikos A., Patil B.S., Deyhim F. (2011). Drinking Carrot Juice Increases Total Antioxidant Status and Decreases Lipid Peroxidation in Adults. Nutr. J..

[54] Kim M., Hwang S., Park E., Bae J. (2013). Strict Vegetarian Diet Improves the Risk Factors Associated with Metabolic Diseases by Modulating Gut Microbiota and Reducing Intestinal Inflammation. Environ. Microbiol. Rep..

[55] Buscemi S., Rosafio G., Arcoleo G., Mattina A., Canino B., Montana M., Verga S., Rini G. (2012). Effects of Red Orange Juice Intake on Endothelial Function and Inflammatory Markers in Adult Subjects with Increased Cardiovascular Risk. Am. J. Clin. Nutr..

[56] Mirmiran P. (2014). Functional Foods-Based Diet as a Novel Dietary Approach for Management of Type 2 Diabetes and Its Complications: A Review. World J. Diabetes.

[57] Alkhatib A., Tsang C., Tiss A., Bahorun T., Arefanian H., Barake R., Khadir A., Tuomilehto J. (2017). Functional Foods and Lifestyle Approaches for Diabetes Prevention and Management. Nutrients.

[58] Smith L., López Sánchez G.F., Veronese N., Soysal P., Oh H., Barnett Y., Keyes H., Butler L., Allen P., Kostev K. (2022). Fruit and Vegetable Intake and Non-Communicable Diseases among Adults Aged ≥50 Years in Low- and Middle-Income Countries. J. Nutr. Health Aging.

[59] Zheng J., Zhou Y., Li S., Zhang P., Zhou T., Xu D.-P., Li H.-B. (2017). Effects and Mechanisms of Fruit and Vegetable Juices on Cardiovascular Diseases. Int. J. Mol. Sci..

[60] Afshin A., Sur P.J., Fay K.A., Cornaby L., Ferrara G., Salama J.S., Mullany E.C., Abate K.H., Abbafati C., Abebe Z. (2019). Health Effects of Dietary Risks in 195 Countries, 1990–2017: A Systematic Analysis for the Global Burden of Disease Study 2017. Lancet.

[61] FAO (2004). WHO Fruit and Vegetables for Health. Rep. a Jt. FAO WHO Work..

[62] Li M., Fan Y., Zhang X., Hou W., Tang Z. (2014). Fruit and Vegetable Intake and Risk of Type 2 Diabetes Mellitus: Meta-Analysis of Prospective Cohort Studies. BMJ Open.

[63] Anderson R.A., Broadhurst C.L., Polansky M.M., Schmidt W.F., Khan A., Flanagan V.P., Schoene N.W., Graves D.J. (2004). Isolation and Characterization of Polyphenol Type-A Polymers from Cinnamon with Insulin-like Biological Activity. J. Agric. Food Chem..

[64] Babbar N., Oberoi H.S., Sandhu S.K., Bhargav V.K. (2014). Influence of Different Solvents in Extraction of Phenolic Compounds from Vegetable Residues and Their Evaluation as Natural Sources of Antioxidants. J. Food Sci. Technol..

[65] Lin D., Xiao M., Zhao J., Li Z., Xing B., Li X., Kong M., Li L., Zhang Q., Liu Y. (2016). An Overview of Plant Phenolic Compounds and Their Importance in Human Nutrition and Management of Type 2 Diabetes. Molecules.

[66] Survay N.S., Ko E., Upadhyay C.P., Mi J., Park S.W., Lee D., Jung Y.-S., Yoon D.-Y., Hong S. (2010). Hypoglycemic Effects of Fruits and Vegetables in Hyperglycemic Rats for Prevention of Type-2 Diabetes. Korean J. Hortic. Sci. Technol..

[67] Jayaprakasam B., Vareed S.K., Olson L.K., Nair M.G. (2005). Insulin Secretion by Bioactive Anthocyanins and Anthocyanidins Present in Fruits. J. Agric. Food Chem..

[68] Wedick N.M., Pan A., Cassidy A., Rimm E.B., Sampson L., Rosner B., Willett W., Hu F.B., Sun Q., Van Dam R.M. (2012). Dietary Flavonoid Intakes and Risk of Type 2 Diabetes in US Men and Women. Am. J. Clin. Nutr..

[69] Mazlan F.A., Suffian M., Sharifuddin Y. (2015). Biotransformation of Momordica Charantia Fresh Juice by Lactobacillus Plantarum BET003 and Its Putative Anti-Diabetic Potential. PeerJ.

[70] Badejo A.A., Falarunu A.J., Duyilemi T.I., Fasuhanmi O.S. (2020). Antioxidative and Anti-Diabetic Potentials of Tigernut (*Cyperus esculentus*) Sedge Beverages Fortified with Vernonia Amygdalina and Momordica Charantia. J. Food Meas. Charact..

[71] Nowicka P., Wojdyło A., Samoticha J. (2016). Evaluation of Phytochemicals, Antioxidant Capacity, and Antidiabetic Activity of Novel Smoothies from Selected Prunus Fruits. J. Funct. Foods.

[72] Ujiroghene O.J., Liu L., Zhang S., Lu J., Zhang C., Pang X., Lv J. (2019). Potent α-Amylase Inhibitory Activity of Sprouted Quinoa-Based Yoghurt Beverages Fermented with Selected Anti-Diabetic Strains of Lactic Acid Bacteria. RSC Adv..

[73] Zhong H., Abdullah, Zhao M., Tang J., Deng L., Feng F. (2021). Probiotics-Fermented Blueberry Juices as Potential Antidiabetic Product: Antioxidant, Antimicrobial and Antidiabetic Potentials. J. Sci. Food Agric..

[74] Etxeberria U., De La Garza A.L., Campin J., Martnez J.A., Milagro F.I. (2012). Antidiabetic Effects of Natural Plant Extracts via Inhibition of Carbohydrate Hydrolysis Enzymes with Emphasis on Pancreatic Alpha Amylase. Expert Opin. Ther. Targets.

[75] Rubilar M., Jara C., Poo Y., Acevedo F., Gutierrez C., Sineiro J., Shene C. (2011). Extracts of Maqui (*Aristotelia chilensis*) and Murta (*Ugni molinae* Turcz.): Sources of Antioxidant Compounds and *α*-Glucosidase/*α*-Amylase Inhibitors. J. Agric. Food Chem..

[76] Wang S., Li Y., Huang D., Chen S., Xia Y., Zhu S. (2022). The Inhibitory Mechanism of Chlorogenic Acid and Its Acylated Derivatives on *α*-Amylase and *α*-Glucosidase. Food Chem..

[77] Nazir N., Zahoor M., Ullah R., Ezzeldin E., Mostafa G.A.E. (2020). Curative Effect of Catechin Isolated from *Elaeagnus umbellata* Thunb. Berries for Diabetes and Related Complications in Streptozotocin-Induced Diabetic Rats Model. Molecules.

[78] Costamagna M.S., Zampini I.C., Alberto M.R., Cuello S., Torres S., Pérez J., Quispe C., Schmeda-Hirschmann G., Isla M.I. (2016). Polyphenols Rich Fraction from *Geoffroea decorticans* Fruits Flour Affects Key Enzymes Involved in Metabolic Syndrome, Oxidative Stress and Inflammatory Process. Food Chem..

[79] Batubara I., Kuspradini H., Muddathir A.M., Mitsunaga T. (2014). *Intsia palembanica* Wood Extracts and Its Isolated Compounds as Propionibacterium Acnes Lipase Inhibitor. J. Wood Sci..

[80] Dalar A., Türker M., Zabaras D., Konczak I. (2014). Phenolic Composition, Antioxidant and Enzyme Inhibitory Activities of *Eryngium bornmuelleri* Leaf. Plant Foods Hum. Nutr..

[81] Mahmoud M.F., El Ashry F.E.Z.Z., El Maraghy N.N., Fahmy A. (2017). Studies on the Antidiabetic Activities of *Momordica charantia* Fruit Juice in Streptozotocin-Induced Diabetic Rats. Pharm. Biol..

[82] Vhora N., Naskar U., Hiray A., Kate A.S., Jain A. (2020). Recent Advances In In-Vitro Assays for Type 2 Diabetes Mellitus: An Overview. Rev. Diabet. Stud..

[83] Zhu C., Lü H., Du L., Li J., Chen H., Zhao H., Wu W., Chen J., Li W. (2023). Five Blueberry Anthocyanins and Their Antioxidant, Hypoglycemic, and Hypolipidemic Effects In Vitro. Front. Nutr..

[84] Castro-Acosta M.L., Stone S.G., Mok J.E., Mhajan R.K., Fu C.I., Lenihan-Geels G.N., Corpe C.P., Hall W.L. (2017). Apple and Blackcurrant Polyphenol-Rich Drinks Decrease Postprandial Glucose, Insulin and Incretin Response to a High-Carbohydrate Meal in Healthy Men and Women. J. Nutr. Biochem..

[85] Wang H.-Y., Kan W.-C., Cheng T.-J., Yu S.-H., Chang L.-H., Chuu J.-J. (2014). Differential Anti-Diabetic Effects and Mechanism of Action of Charantin-Rich Extract of Taiwanese *Momordica charantia* between Type 1 and Type 2 Diabetic Mice. Food Chem. Toxicol..

[86] Tian M., Zeng X.-Q., Song H.-L., Hu S.-X., Wang F.-J., Zhao J., Hu Z.-B. (2015). Molecular Diversity and Hypoglycemic Polypeptide-P Content of *Momordica charantia* in Different Accessions and Different Seasons. J. Sci. Food Agric..

[87] Mahwish, Saeed F., Sultan M.T., Riaz A., Ahmed S., Bigiu N., Amarowicz R., Manea R. (2021). Bitter Melon (*Momordica charantia* L.) Fruit Bioactives Charantin and Vicine Potential for Diabetes Prophylaxis and Treatment. Plants.

[88] Xiao G., Xiao X. (2021). Antidiabetic Effect of Hydro-Methanol Extract of *Prunus cerasus* L Fruits and Identification of Its Bioactive Compounds. Trop. J. Pharm. Res..

[89] Koprivica M.R., Trifković J.Đ., Dramićanin A.M., Gašić U.M., Akšić M.M.F., Milojković-Opsenica D.M. (2018). Determination of the Phenolic Profile of Peach (*Prunus persica* L.) Kernels Using UHPLC–LTQ OrbiTrap MS/MS Technique. Eur. Food Res. Technol..

[90] Iglesias-Carres L., Mas-Capdevila A., Bravo F.I., Bladé C., Arola-Arnal A., Muguerza B. (2019). Optimization of Extraction Methods for Characterization of Phenolic Compounds in Apricot Fruit (*Prunus armeniaca*). Food Funct..

[91] Mihailović N.R., Mihailović V.B., Kreft S., Ćirić A.R., Joksović L.G., Đurđević P.T. (2018). Analysis of Phenolics in the Peel and Pulp of Wild Apples (*Malus sylvestris* (L.) Mill.). J. Food Compos. Anal..

[92] Vagiri M., Ekholm A., Andersson S.C., Johansson E., Rumpunen K. (2012). An Optimized Method for Analysis of Phenolic Compounds in Buds, Leaves, and Fruits of Black Currant (*Ribes nigrum* L.). J. Agric. Food Chem..

[93] Pandya A., Thiele B., Köppchen S., Zurita-Silva A., Usadel B., Fiorani F. (2023). Characterization of Bioactive Phenolic Compounds in Seeds of Chilean Quinoa (*Chenopodium quinoa* Willd.) Germplasm. Agronomy.

[94] Mohamed M., Soliman A., Abbas M., Abd el-lateaf H., Ismael A. (2021). Comparative Study for Physico-Chemical Characteristics of Crude *Moringa Peregrina*, *Terminalia bellerica* and Tiger Nut Oils. Egypt. J. Chem..

[95] Varo M.Á., Martín-Gómez J., Mérida J., Serratosa M.P. (2021). Bioactive Compounds and Antioxidant Activity of Highbush Blueberry (*Vaccinium corymbosum*) Grown in Southern Spain. Eur. Food Res. Technol..

[96] Pop R.M., Socaciu C., Pintea A., Buzoianu A.D., Sanders M.G., Gruppen H., Vincken J. (2013). UHPLC/PDA–ESI/MS Analysis of the Main Berry and Leaf Flavonol Glycosides from Different Carpathian *Hippophaë rhamnoides* L. Varieties. Phytochem. Anal..

[97] Guo R., Guo X., Li T., Fu X., Liu R.H. (2017). Comparative Assessment of Phytochemical Profiles, Antioxidant and Antiproliferative Activities of Sea Buckthorn (*Hippophaë rhamnoides* L.) Berries. Food Chem..

[98] Teleszko M., Wojdyło A., Rudzińska M., Oszmiański J., Golis T. (2015). Analysis of Lipophilic and Hydrophilic Bioactive Compounds Content in Sea Buckthorn (*Hippophaë rhamnoides* L.) Berries. J. Agric. Food Chem..

[99] Nambiar S.S., Paramesha M., Shetty N.P. (2015). Comparative Analysis of Phytochemical Profile, Antioxidant Activities and Foam Prevention Abilities of Whole Fruit, Pulp and Seeds of *Emblica officinalis*. J. Food Sci. Technol..

[100] Almeida É.S., de Oliveira D., Hotza D. (2019). Properties and Applications of *Morinda citrifolia* (Noni): A Review. Compr. Rev. Food Sci. Food Saf..

[101] Ali M.Y., Sina A.A.I., Khandker S.S., Neesa L., Tanvir E.M., Kabir A., Khalil M.I., Gan S.H. (2021). Nutritional Composition and Bioactive Compounds in Tomatoes and Their Impact on Human Health and Disease: A Review. Foods.

[102] Banihani S., Swedan S., Alguraan Z. (2013). Pomegranate and Type 2 Diabetes. Nutr. Res..

[103] Ballistreri G., Fabroni S., Romeo F.V., Timpanaro N., Amenta M., Rapisarda P. (2019). Anthocyanins and Other Polyphenols in Citrus Genus: Biosynthesis, Chemical Profile, and Biological Activity. Polyphenols in Plants.

[104] Cai R., Hettiarachchy N.S., Jalaluddin M. (2003). High-Performance Liquid Chromatography Determination of Phenolic Constituents in 17 Varieties of Cowpeas. J. Agric. Food Chem..

[105] Salas-Arias K., Irías-Mata A., Sánchez-Kopper A., Hernández-Moncada R., Salas-Morgan B., Villalta-Romero F., Calvo-Castro L.A. (2023). *Strawberry Fragaria x ananassa* Cv. Festival: A Polyphenol-Based Phytochemical Characterization in Fruit and Leaf Extracts. Molecules.

[106] Šedbarė R., Siliņa D., Janulis V. (2022). Evaluation of the Phytochemical Composition of Phenolic and Triterpene Compounds in Fruit of Large Cranberries (*Vaccinium macrocarpon* Aiton) Grown in Latvia. Plants.

[107] Pacheco-Palencia L.A., Mertens-Talcott S., Talcott S.T. (2008). Chemical Composition, Antioxidant Properties, and Thermal Stability of a Phytochemical Enriched Oil from Açai (*Euterpe oleracea* Mart.). J. Agric. Food Chem..

[108] Cebadera L., Dias M.I., Barros L., Fernández-Ruiz V., Cámara R.M., Del Pino Á., Santos-Buelga C., Ferreira I.C.F.R., Morales P., Cámara M. (2020). Characterization of Extra Early Spanish Clementine Varieties (*Citrus clementina* Hort Ex Tan) as a Relevant Source of Bioactive Compounds with Antioxidant Activity. Foods.

[109] Gargi B., Semwal P., Jameel Pasha S.B., Singh P., Painuli S., Thapliyal A., Cruz-Martins N. (2022). Revisiting the Nutritional, Chemical and Biological Potential of *Cajanus cajan* (L.) Millsp. Molecules.

[110] Kottaisamy C.P.D., Raj D.S., Prasanth Kumar V., Sankaran U. (2021). Experimental Animal Models for Diabetes and Its Related Complications—A Review. Lab. Anim. Res..

[111] Guo Y., Wu G., Su X., Yang H., Zhang J. (2009). Antiobesity Action of a Daidzein Derivative on Male Obese Mice Induced by a High-Fat Diet. Nutr. Res..

[112] Nerurkar P.V., Nishioka A., Eck P.O., Johns L.M., Volper E., Nerurkar V.R. (2012). Regulation of Glucose Metabolism via Hepatic forkhead Transcription Factor 1 (FoxO1) by *Morinda citrifolia* (noni) in High-Fat Diet-Induced Obese Mice. Br. J. Nutr..

[113] Seo K., Lee J., Choi R., Lee H.-I., Lee J.-H., Jeong Y., Kim M., Lee M. (2014). Anti-Obesity and Anti-Insulin Resistance Effects of Tomato Vinegar Beverage in Diet-Induced Obese Mice. Food Funct..

[114] Chudnovskiy R., Thompson A., Tharp K., Hellerstein M., Napoli J.L., Stah A. (2014). Consumption of Clarified Grapefruit Juice Ameliorates High-Fat Diet Induced Insulin Resistance and Weight Gain in Mice. PLoS ONE.

[115] Variya B.C., Bakrania A.K., Patel S.S. (2020). Antidiabetic Potential of Gallic Acid from *Emblica officinalis*: Improved Glucose Transporters and Insulin Sensitivity through PPAR-γ and Akt Signaling. Phytomedicine.

[116] Fukaya M., Sato Y., Kondo S., Adachi S.I., Yoshizawa F., Sato Y. (2021). Quercetin enhances fatty acid β-oxidation by inducing lipophagy in AML12 hepatocytes. Heliyon.

[117] van Raalte D.H., Li M., Pritchard P.H., Wasan K.M. (2004). Peroxisome Proliferator-Activated Receptor (PPAR)-: A Pharmacological Target with a Promising Future. Pharm. Res..

[118] Ariviani S., Affandi D.R., Listyaningsih E., Handajani S. (2018). The Potential of Pigeon Pea (*Cajanus cajan*) Beverage as an Anti-diabetic Functional Drink. IOP Conf. Ser. Earth Environ. Sci..

[119] Bolsinger J., Pronczuk A., Sambanthamurthi R., Hayes K.C. (2014). Anti-Diabetic Effects of Palm Fruit Juice in the Nile Rat (*Arvicanthis niloticus*). J. Nutr. Sci..

[120] Gao H., Wen J.-J., Hu J.-L., Nie Q.-X., Chen H.H., Xiong T., Nie S.-P., Xie M.-Y. (2019). Fermented *Momordica charantia* L. Juice Modulates Hyperglycemia, Lipid Profile, and Gut Microbiota in Type 2 Diabetic Rats. Food Res. Int..

[121] Iwansyah A.C., Luthfiyanti R., Ardiansyah R.C.E., Rahman N., Andriana Y., Hamid H.A. (2022). Antidiabetic Activity of *Physalis angulata* L. Fruit juIce on Streptozotocin-Induced Diabetic Rats. S. Afr. J. Bot..

[122] Hu J., Nie S., Xie M. (2018). Antidiabetic Mechanism of Dietary Polysaccharides Based on Their Gastrointestinal Functions. J. Agric. Food Chem..

[123] Association A.D. (2009). Diagnosis and Classification of Diabetes Mellitus. Diabetes Care.

[124] Shi G.-J., Li Y., Cao Q.-H., Wu H.-X., Tang X.-Y., Gao X.-H., Yu J.-Q., Chen Z., Yang Y. (2019). In Vitro and In Vivo Evidence That Quercetin Protects against Diabetes and Its Complications: A Systematic Review of the Literature. Biomed. Pharmacother..

[125] Youl E., Bardy G., Magous R., Cros G., Sejalon F., Virsolvy A., Richard S., Quignard J.F., Gross R., Petit P. (2010). Quercetin Potentiates Insulin Secretion and Protects INS-1 Pancreatic-Cells against Oxidative Damage via the ERK1/2 Pathway. Br. J. Pharmacol..

[126] Adewole S., Caxton-Martins E., Ojewole J. (2007). Protective Effect of Quercetin on the Morphology of Pancreatic *β*-Cells of Streptozotocin-Treated Diabetic Rats. Afr. J. Tradit. Complement. Altern. Med..

[127] Fang X.K., Gao J., Zhu D.N. (2008). Kaempferol and Quercetin Isolated from *Euonymus alatus* Improve Glucose Uptake of 3T3-L1 Cells without Adipogenesis Activity. Life Sci..

[128] Guo X., Zhang D., Gao X., Parry J., Liu K., Liu B., Wang M. (2013). Quercetin and Quercetin-3-O-Glucuronide Are Equally Effective in Ameliorating Endothelial Insulin Resistance through Inhibition of Reactive Oxygen Species-Associated Inflammation. Mol. Nutr. Food Res..

[129] Soltesova-Prnova M., Milackova I., Stefek M. (2016). 3′-O-(3-Chloropivaloyl)Quercetin, *α*-Glucosidase Inhibitor with Multi-Targeted Therapeutic Potential in Relation to Diabetic Complications. Chem. Pap..

[130] Kamiya K., Hamabe W., Harada S., Murakami R., Tokuyama S., Satake T. (2008). Chemical Constituents of *Morinda citrifolia* Roots Exhibit Hypoglycemic Effects in Streptozotocin-Induced Diabetic Mice. Biol. Pharm. Bull..

[131] O’Neill H.M. (2013). AMPK and Exercise: Glucose Uptake and Insulin Sensitivity. Diabetes Metab.

[132] Prabhakar P.K., Doble M. (2011). Mechanism of Action of Natural Products Used in the Treatment of Diabetes Mellitus. Chin. J. Integr. Med..

[133] Pitipanapong J., Chitprasert S., Goto M., Jiratchariyakul W., Sasaki M., Shotipruk A. (2007). New Approach for Extraction of Charantin from *Momordica charantia* with Pressurized Liquid Extraction. Sep. Purif. Technol..

[134] Eze E.D., Tanko Y., Tende J.A., Ehinomhen U.A. (2016). Effects of Lycopene on Liver Markers and Glucokinase Activity in Experimentally-induced Diabetes Mellitus Rat Model. J. Basic Appl. Res.

[135] Huang S., Czech M.P. (2007). The *GLUT4* Glucose Transporter. Cell Metab..

[136] Kouznetsova V.L., Hauptschein M., Tsigelny I.F. (2017). Glucose and Lipid Transporters Roles in Type 2 Diabetes. Integr. Obes. Diabetes.

[137] Wang D., Yan J., Chen J., Wu W., Zhu X., Wang Y. (2015). Naringin Improves Neuronal Insulin Signaling, Brain Mitochondrial Function, and Cognitive Function in High-Fat Diet-Induced Obese Mice. Cell. Mol. Neurobiol..

[138] Leow S., Bolsinger J., Pronczuk A., Hayes K.C., Sambanthamurthi R. (2016). Hepatic Transcriptome Implications for Palm Fruit Juice Deterrence of Type 2 Diabetes Mellitus in Young Male Nile Rats. Genes Nutr..

[139] Ormazabal V., Nair S., Elfeky O., Aguayo C., Salomon C., Zuñiga F.A. (2018). Association between Insulin Resistance and the Development of Cardiovascular Disease. Cardiovasc. Diabetol..

[140] Goldstein B.J. (2002). Insulin Resistance as the Core Defect in Type 2 Diabetes Mellitus. Am. J. Cardiol..

[141] Vergès B. (2015). Pathophysiology of Diabetic Dyslipidaemia: Where Are We?. Diabetologia.

[142] Dikshit P., Shukla K., Tyagi M.K., Garg P., Gambhir J.K., Shukla R. (2012). Antidiabetic and Antihyperlipidemic Effects of the Stem of *Musa sapientum* Linn. in Streptozotocin-Induced Diabetic Rats. J. Diabetes.

[143] Koebnick C., Garcia A.L., Dagnelie P.C., Strassner C., Lindemans J., Katz N., Leitzmann C., Hoffmann I. (2005). Long-Term Consumption of a Raw Food Diet is Associated with Favorable Serum LDL Cholesterol and Triglycerides But Also with Elevated Plasma Homocysteine and Low Serum HDL Cholesterol in Humans. J. Nutr..

[144] Ullah A., Khan A., Khan I. (2016). Diabetes Mellitus and Oxidative Stress—A Concise Review. Saudi Pharm. J..

[145] Banihani S.A., Makahleh S.M., El-Akawi Z., Al-Fashtaki R.A., Khabour O.F., Gharibeh M.Y., Saadah N.A., Al-Hashimi F.H., Al-Khasieb N.J. (2014). Fresh Pomegranate Juice Ameliorates Insulin Resistance, Enhances β-Cell Function, and Decreases Fasting Serum Glucose in Type 2 Diabetic Patients. Nutr. Res..

[146] Hontecillas R., O’Shea M., Einerhand A., Diguardo M., Bassaganya-Riera J. (2009). Activation of PPAR γ and *α* by Punicic Acid Ameliorates Glucose Tolerance and Suppresses Obesity-Related Inflammation. J. Am. Coll. Nutr..

[147] Koren-Gluzer M., Aviram M., Meilin E., Hayek T. (2011). The Antioxidant HDL-Associated Paraoxonase-1 (PON1) Attenuates Diabetes Development and Stimulates *β*-Cell Insulin Release. Atherosclerosis.

[148] Devaki C.S., Premavalli K.S. (2014). Evaluation of Supplementation of Bittergourd Fermented Beverage to Diabetic Subjects. J. Pharm. Nutr. Sci..

[149] Paquette M., Medina Larqué A.S., Weisnagel S.J., Desjardins Y., Marois J., Pilon G., Dudonné S., Marette A., Jacques H. (2017). Strawberry and Cranberry Polyphenols Improve Insulin Sensitivity in Insulin-Resistant, Non-Diabetic Adults: A Parallel, Double-Blind, Controlled and Randomised Clinical Trial. Br. J. Nutr..

[150] Kim H., Simbo S.Y., Fang C., McAlister L., Roque A., Banerjee N., Talcott S.T., Zhao H., Kreider R.B., Mertens-Talcott S.U. (2018). Açaí (*Euterpe oleracea* Mart.) Beverage Consumption Improves Biomarkers for Inflammation but Not Glucose- or Lipid-Metabolism in Individuals with Metabolic Syndrome in a Randomized, Double-Blinded, Placebo-Controlled Clinical Trial. Food Funct..

[151] Aktan A., Ozcelik A., Cure E., Cure M., Yuce S. (2014). Profound Hypoglycemia-Induced by *Vaccinium corymbosum* Juice and *Laurocerasus* Fruit. Indian J. Pharmacol..

[152] Hasniyati R., Yuniritha E., Fadri R.A. The Efficacy of Therapeutic-Diabetes Mellitus Functional Drink on Blood Glucose and Plasma Malondialdehyde (MDA) Levels of Type 2 Diabetes Mellitus Patients. Proceedings of the 1st Lekantara Annual Conference on Natural Science and Environment (LeNS).

[153] Sequeira I., Poppitt S. (2017). Unfolding Novel Mechanisms of Polyphenol Flavonoids for Better Glycaemic Control: Targeting Pancreatic Islet Amyloid Polypeptide (IAPP). Nutrients.

[154] Li D., Zhang Y., Liu Y., Sun R., Xia M. (2015). Purified Anthocyanin Supplementation Reduces Dyslipidemia, Enhances Antioxidant Capacity, and Prevents Insulin Resistance in Diabetic Patients. J. Nutr..

[155] Cladis D.P., Li S., Reddivari L., Cox A., Ferruzzi M.G., Weaver C.M. (2020). A 90 Day Oral Toxicity Study of Blueberry Polyphenols in Ovariectomized Sprague-Dawley Rats. Food Chem. Toxicol..

[156] Cladis D.P., Weaver C.M., Ferruzzi M.G. (2022). (Poly)Phenol Toxicity In Vivo Following Oral Administration: A Targeted Narrative Review of (Poly)Phenols from Green Tea, Grape, and Anthocyanin-Rich Extracts. Phyther. Res..

[157] Patel S.S., Goyal R.K. (2011). Prevention of Diabetes-Induced Myocardial Dysfunction in Rats Using the Juice of the *Emblica officinalis* Fruit. Exp. Clin. Cardiol..

[158] Dhuique-mayer C., Gence L., Portet K., Tousch D., Poucheret P. (2020). Preventive Action of Retinoids in Metabolic Syndrome/Type 2 Diabetic Rats Fed with Citrus Functional Food Enriched in β-Cryptoxanthin Claudie. Food Funct..

[159] Zhou Y., Zheng J., Li S., Zhou T., Zhang P., Li H. (2016). Bin Alcoholic Beverage Consumption and Chronic Diseases. Int. J. Environ. Res. Public Health.

[160] Conigrave K.M., Rimm E.B. (2003). Alcohol for the Prevention of Type 2 Diabetes Mellitus?. Treat. Endocrinol..

[161] Sanz M. (2004). Inositols and Carbohydrates in Different Fresh Fruit Juices. Food Chem..

[162] Lifschitz C.H. (2000). Carbohydrate Absorption From Fruit Juices in Infants. Pediatrics.

[163] Bazzano L.A., Li T.Y., Joshipura K.J., Hu F.B. (2008). Intake of Fruit, Vegetables, and Fruit Juices and Risk of Diabetes in Women. Diabetes Care.

